# Ultrasensitive biosensors based on waveguide-coupled long-range surface plasmon resonance (WC-LRSPR) for enhanced fluorescence spectroscopy†

**DOI:** 10.1039/d1ra02130c

**Published:** 2021-06-25

**Authors:** Nhu Hoa Thi Tran, Viet-Duc Phung, Hanh Kieu Thi Ta, Vu Dinh Lam, Do Hung Manh, Ngoc Kim Pham, Jae Young Kim, Nae Yoon Lee, Bach Thang Phan

**Affiliations:** Faculty of Materials Science, University of Science HoChiMinh City Vietnam ttnhoa@hcmus.edu.vn; Vietnam National University HoChiMinh City Vietnam; Future Materials and Devices Laboratory, Duy Tan University Ho Chi Minh City 700000 Vietnam; Faculty of Environmental and Chemical Engineering, Duy Tan University Da Nang 550000 Vietnam; Center for Innovative Materials and Architectures (INOMAR) HoChiMinh City Vietnam pbthang@inomar.edu.vn; Graduate University of Science and Technology, Vietnam Academy of Science and Technology Hanoi Vietnam; Institute of Materials Science, Vietnam Academy of Science and Technology Hanoi Vietnam; Department of Life Science, Gachon University Seongnam-si Gyeonggi-do 13120 Republic of Korea; Department of BioNano Technology, Gachon University Seongnam-si Gyeonggi-do 13120 Republic of Korea; Laboratory of Advanced Materials, University of Science HoChiMinh City Vietnam

## Abstract

We investigated the coupling phenomenon between plasmonic resonance and waveguide modes through theoretical and experimental parametric analyses on the bimetallic waveguide-coupled long-range surface plasmon resonance (Bi-WCLRSPR) structure. The calculation results indicated that the multi-plasmonic coupling gives rise to the enhanced depth-to-width ratio of the reflection dip compared to that of LRSPR excited using a single set of Ag and Teflon. The optimized thickness of Ag(40 nm)/Teflon(700 nm)/Ag(5 nm)/Au(5 nm) was obtained and generated the highest plasmon intensity enhancement, which was 2.38 folds in comparison to the conventional bimetallic surface plasmon resonance (SPR) configuration (Ag/Au). 17β-Estradiol was used in the fluorescence enhancement experiment by the reflection geometry-based system, wherein the excitation light source was on the side of a WC-LRSPR chip opposite to that of the light detection unit. The phenomenon of surface plasmon-couple emission (SPCE) depends on the number of 17β-estradiol molecule promoters from female sex steroid hormones, which demonstrated a limit of detection (LOD) of 2 pg mL^−1^ and 1.47-fold fluorescence improvement as compared to the non-coated material on the surface of pristine glass. This enhanced WC-LRSPR can readily find application in fluorescence escalation needed in cases where a weak fluorescence signal is predicted, such as the small volume of liquid containing fluorescent dyes in biological diagnosis.

## Abstract 2

We investigated the coupling phenomenon between plasmonic resonance and waveguide modes through theoretical and experimental parametric analyses on the bimetallic waveguide-coupled long-range surface plasmon resonance (Bi-WCLRSPR) structure.

## Graphical abstract

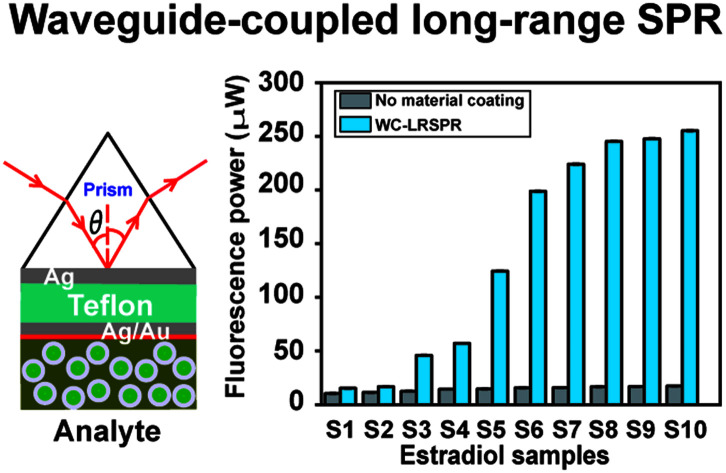

## Introduction

The phenomenon of SPR sensors has been exploited for a long time and has been used for food quality tests,^1–3^ drug development,^4,5^ medical diagnostics,^6–8^ and biomedical applications^9,10^ due to the advantages of biomolecular interactions in real-time, and relatively high sensitivity of label-free biosensing techniques.^11,12^ Surface-plasmons are bound electromagnetic waves that propagate along with the interface of two materials with real dielectric constants of opposite signs. SPR is an optical excitation of a surface wave due to an oscillation of charge density at the boundary between two different media with the permittivity of opposite signs such as metals and dielectrics.^13,14^ Resonance condition is established when the wave vector of the evanescent field of light used matches with that of surface plasmon wave at an interface with the specific angle of the incident light. Silver and gold are generally used for the metal layer in SPR sensors because of their stability. Moreover, they possess negative real relative permittivities opposite to the positive real dielectric constants of the dielectric media.^15–17^ In general, the conventional method used the gold (Au) film SPR-sensing based on the prism Kretschmann configuration^18^ because of the highest sensitivity; however, the resolution is the lowest when comparing the Au, silver (Ag), or bimetallic (Au–Ag) films.^19,20^ Because of the chemical instability of Ag, their combination in silver (Ag)–gold (Au) bimetallic nanofilm is advantageous in an SPR optical biosensor. The electric field (E-field) enhancement factor at the metal–analyte interface of a bimetallic nanofilm is also greater than that for Au.^19^ The excitation of surface plasmons based on the prism can be broadly categorized namely as the operation of SPR-based, LRSPR,^21,22^ coupled plasmon-waveguide resonance (CPWR),^23–25^ and waveguide-coupled SPR (WCSPR).^26^ Another feasible approach to decrease the full width at half maximum (FWHM) or increase the sensitivity is to use coupled surface plasma modes,^27,28^ particularly LRSPR and WCSPR, which not only maintain the high sensitivity of conventional SPR sensors but also yield an extremely narrow FWHM. The LRSPR structure, comprising a thin metal layer sandwiched between two insulators, was first proposed by Sarid in 1981.^29^ Quail *et al.* observed the LRSPR phenomenon experimentally on Ag and aluminium (Al) films, proving that the LRSPR can reduce the resonance-angle width by an order of magnitude compared with conventional SPR sensors.^21^ It was initially thought that only a symmetric structure could excite the LRSPR; however, multilayer asymmetric LRSPR geometries have been introduced that exhibit great potential for high-performance LRSPR sensors.^30–32^ In our previous work related to LRSPR, we have tried to characterize the dielectric material from the progressive nature of the multiple resonance peaks in a multilayer composite plasmonic structure.^33^

As a complementary geometry to the insulator–metal–insulator (IMI) LRSPR geometry, the WC-LRSPR structure comprises a dielectric waveguide layer embedded in two metallic layers. It has important characteristics, *e.g.*, it can be operated in both transverse-electric (TE) and transverse-magnetic (TM) polarized waves and exhibit multi-peaks that allow investigating the dielectric constant and the thickness of self-assembled monolayers and thin films.^25,34^ Combining the SPR and waveguide modes in the WC-LRSPR geometry not only retains the sensitivity of the sensors but also generates a deep and sharp resonance dip, enhancing the detection accuracy of the resonance-angle position.^35,36^ This structure provides multiple resonance peaks in WC-LRSPR dips to increase the sensitivity of sensors and for some other bio-photonic applications.^36^ Although there is abundant literature concerning the properties of the aforementioned configurations, few reports directly compare the signal-to-noise ratio (SNR) among them, especially for bimetallic SPR modes.

Estradiol (E_2_), is the natural sex hormone responsible for development of the female reproductive system and secondary sex characteristics.^37^ Exposure to excessive E_2_ can lead to the disruption of endocrine functions.^38^ However, the small quantities of hormones present in blood have complicated its estimation and require complicated technology like enzyme-linked immunosorbent assay (ELISA),^39^ and LC-MS,^40^ with elaborate instrumental setup, multistep, and time-consuming process. SPR optical sensors are one of the most explored optical devices for trace detection in biomedical diagnostics^41^ due to its unique properties such as rapid, high detection sensitivity, miniaturization and point-of-care sensing.^42^ Much of the earlier work has been on the SPR based immunoassay for the screening of estradiol with the detection limits of 25 pg mL^−1^,^43^ 25 pg mL^−1^,^44^ and 100 μg mL^−1^.^45^

In this study, we directly compared the enhancement of *Γ* and FWHM of the dip in the SPR reflectance curves by using four different kinds of SPR modes: long-range SPR for single or bi-layer of the metal, *i.e.*, [prism]–[Teflon]–[Ag] or [prism]–[Teflon]–[Ag]–[Au]; and combining LRSPR and single or bi-layer of the metal waveguide-coupled SPR *i.e.*, [prism]–[Ag1]–[Teflon]–[Ag2] or [prism]–[Ag1]–[Teflon]–[Ag2]–[Au]. Theoretical modeling has been carried out by solving the transfer matrix equations for the multilayer stack of Ag, dielectric waveguide Teflon, Au geometry to improve by decreasing FWHM of the SPR reflectivity dip curves.^46,47^ The thickness of each layer is varied in order to investigate the role of resonance in the coupled plasmonic configuration. Here in this work, the thicknesses of Ag, Teflon, and Au layers were first optimized with respect to high-*Γ*, FWHM, minimum reflectance at the excitation *λ*_ex_ = 498 nm, and electric-field intensity (|*E*|^2^), which were compared to reveal the features. However, to the best of our knowledge, this type of sensor performance with the smallest FWHM, simultaneously, has not been recorded by previous researchers in this field. This study suggests a method for understanding the relative impacts of various SPR modes and hence enables the sensitivity of these configurations to be improved in further studies. Numerical simulation results show that the Ag(40 nm)/Teflon(700 nm)/Ag(5 nm)/Au(5 nm) as the WC-LSPR coating material can extend the characteristic depth over which the SPR evanescent fields decay at *λ*_ex_, increasing the portion of the fluorophores interacting with plasmonic fields at both spectral channels of excitation.

For experimental studies, the reflection configuration presented finds practical use in designing a fluorescence-based assay device with a compact and miniaturized structure in simple reflection geometry, a feature that supports its usefulness in a practical fluorescence setup. We report a dielectric waveguide (Teflon) on the SPR sensor with an outer layer of 5 nm Au for the sensitive detection of 17β-estradiol with sandwich assay interaction. We experimentally achieved an enhancement in fluorescence detection by 1.47-fold at the estradiol concentration of 2 pg mL^−1^ in the reflection mode. Furthermore, we demonstrate the bleaching-free feature, whereby, linear relation is maintained between the estradiol concentration (below 200 pg mL^−1^) and fluorescence intensity. Our result also indicates that a smaller concentration of fluorophores can produce greater efficiency in fluorescence enhancement.

## Theoretical model and design consideration

### Mathematical modeling of reflectivity

In this study, all calculations were performed using a MATLAB-based program (The Mathworks, Inc. Natick, MA) and a simple equation derived from the multilayer Fresnel equation. Three structures of the SPR biosensor were considered: (I-1) long-range SPR for the signal metal layer, (I-2) long-range SPR for bimetallic metal layers, (II-1) combining LRSPR and single waveguide-coupled SPR, and (II-2) combining LRSPR and bimetallic waveguide-coupled SPR. The theoretical simulation study was based on the prism (BK7 glass, *n* = 1.51)-coupled angular modulation with a precision of 0.01°. A p-polarized light source from a LED with *λ*_ex_ = 488 nm was assumed as the incident wavelength light. Teflon (*n* = 1.311)^33,48,49^ was adopted as a dielectric film layer between the prism and the first metal for LRSPR and WCSPR modes because its refractive index is lower than that of prism but similar to water (*n* = 1.33). We had used Ag and Au simultaneously because Ag gives a shaper SPR dip spectrum than Au.^50^ In Bi-LRSPR and Bi-WCSPR structures, Ag was used as the upper metal layer along with Au as the outer layer depending on the oxidation phenomenon problem of Ag will be removed.^50^ A set of parameters of real and imaginary parts of the dielectric functions of Ag and Au metal layers were taken from a database at wavelengths of excitation (*λ*_ex_ = 498 nm).^51^ The numerical approach is based on the excitation of surface plasmon by the attenuated total reflection (ATR) coupler method. A detailed theoretical simulation for the multilayer structure had been reported in our article.^33^ Here, the reflectance of a generalized N-layer can be computed using the multilayer films transfer matrix theory and Fresnel equations.^52,53^ From the SPR wave of the dip in the reflectance curves, we could calculate the quality factor (*Γ*) by a ratio of SPR dip moves and FWHM as the same previous research.^33^ The depth-to-width ratio of the reflectance dip determines the quality factor of the SPR waves and dominates the resolution in SPR sensor applications and SPR evanescent wave penetration depth.^33^ In our previous study, theoretical modeling research was carried out and analysis for such a structure using a transfer matrix approach to improve the FWHM of the dip in an SPR reflectance curve, and also following the theoretical study of Pedrotti *et al.*^52^

### Electric-field intensity

When the SPR phenomenon occurs, the energy of the evanescent wave is absorbed by the surface plasmon wave (SPW), and thus the reflected intensity *R* within the prism side reaches a bottle, and the intensity of the electromagnetic field reaches its maximum value on the metal surface owing to the enhancement by SPW. The maximum value of the enhancement is obtained using the metal–analyte interface, and for an *N*-layer system (layers 0 and *N* refer to the prism and the analyte, respectively) with p-polarized incident light, it can be defined as:^54^1
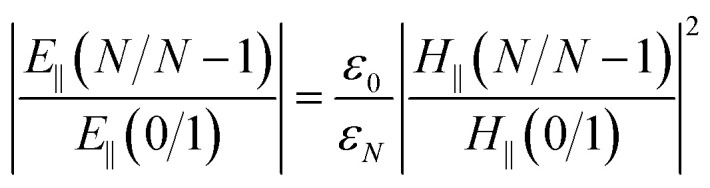
where 
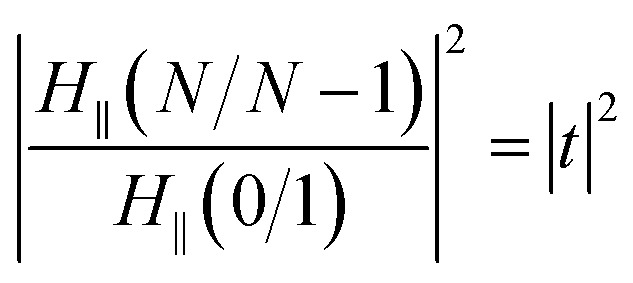
, and *t* is the transmission coefficient of the *N* layer system, as given by eqn (1). *H*_*y*_ is the magnetic field of the SPW, with the *x*-direction assumed as the propagation direction of the SPW, and *E*_∥_ is the electric field of the SPW.

So, the enhancement of the electric field amplitude at the surface of the outer Au layer at *λ*_ex_ was estimated by2
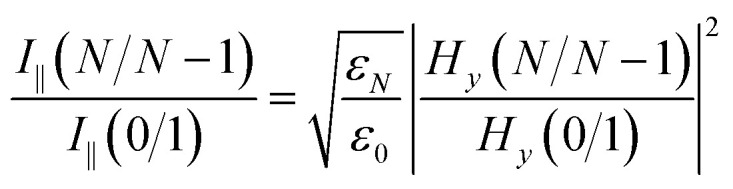


The evanescent-field penetration depth *d* can be considered the distance at which the electric field is reduced to 1/*e* of its initial value3
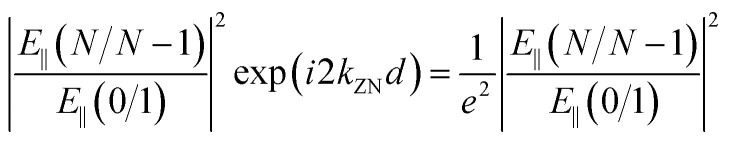
where 
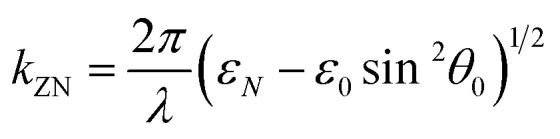
, *θ*_0_ is the resonance angle, and *ε*_0_, *ε*_*N*_ are the dielectric constants of the prism and the *N*^th^-layer medium, respectively.

Therefore, it is essential that the evanescent field, which penetrates into the biological sample, is strong enough to sense the analyte that is bound to the ligand. Since the evanescent field decays exponentially into the biological sample, it is highly desirable that the size of the ligands immobilized on the metal surface is minimized to achieve maximum sensitivity.

## Experimental

### Materials and reagents

Anti-17-estradiol antibody and 17β-estradiol were purchased from Abcam (Cambridge, UK). The Alexa fluor 488-conjugated goat anti-rabbit IgG (H + L) cross-adsorbed secondary antibody was purchased from Thermo Fisher Scientific (Massachusetts, USA). Cysteamine hydrochloride (≥99%), bovine serum albumin (BSA, ≥99%), phosphate-buffered saline (PBS, 1X), (3-aminopropyl)triethoxysilane (APTES, 99%), ethanol (EtOH, 99.8%), polytetrafluoroethylene (PTFE, 99%), perfluoro(*n*-butyltetrahydrofuran) (FC-40, 99%), and 1*H*,1*H*,2*H*,2*H*-perfluorodecyltriethoxysilane (97%) were purchased from Sigma Aldrich. A soda-lime glass substrate was bought from ISOLAB Laborgeräte GmbH. Ag and Au pellets (99.99%) were bought from iTASCO (Taewon Scientific Co., Ltd, Korea). Aqueous solutions were prepared using ultrapure water (Thermo Scientific Easypure II, Göteborg, Sweden) with a resistivity of 18.2 MΩ cm.

### Sensing surface arrangement for immobilization estradiol on the surface

**Fig. 1:**
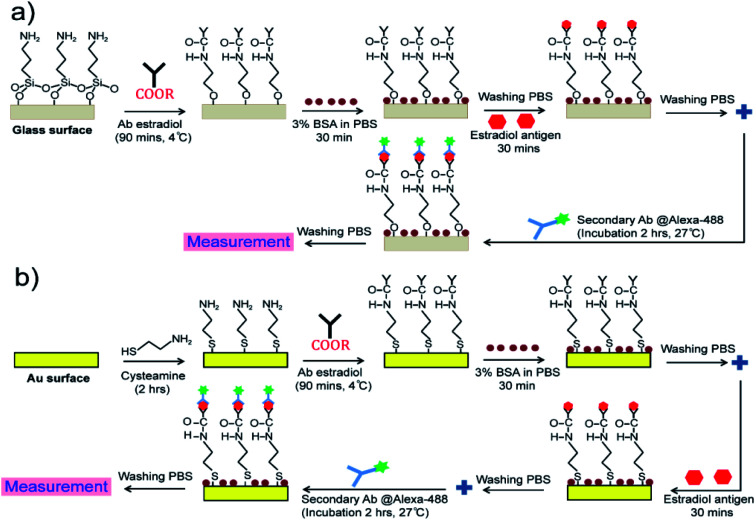
Monoclonal antibody immobilization of estradiol antigens on: (a) glass substrate, and (b) the WC-LRSPR sensor.

Glass and WC-LRSPR with Au thin films on the surfaces were amine-functionalized in 2% APTES and 0.1 M cysteamine solution for 2 h, respectively. Those samples were incubated with the anti-17-β-estradiol antibody (Ab1) solution for 90 min at 4 °C and rinsed with PBS buffer. We used 2% BSA in PBS for blocking buffer before adding the antigen to the samples. The sensor was calibrated in the range of 2–1000 pg mL^−1^ for 17β-estradiol. In about two hours, the samples were incubated in a secondary antibody labeled with Alexa 488 in a humid environment to increase the antibody binding affinity. A schematic of surface functionalization steps is illustrated in Fig. 1.

### Sensor configuration and measurements

**Fig. 2:**
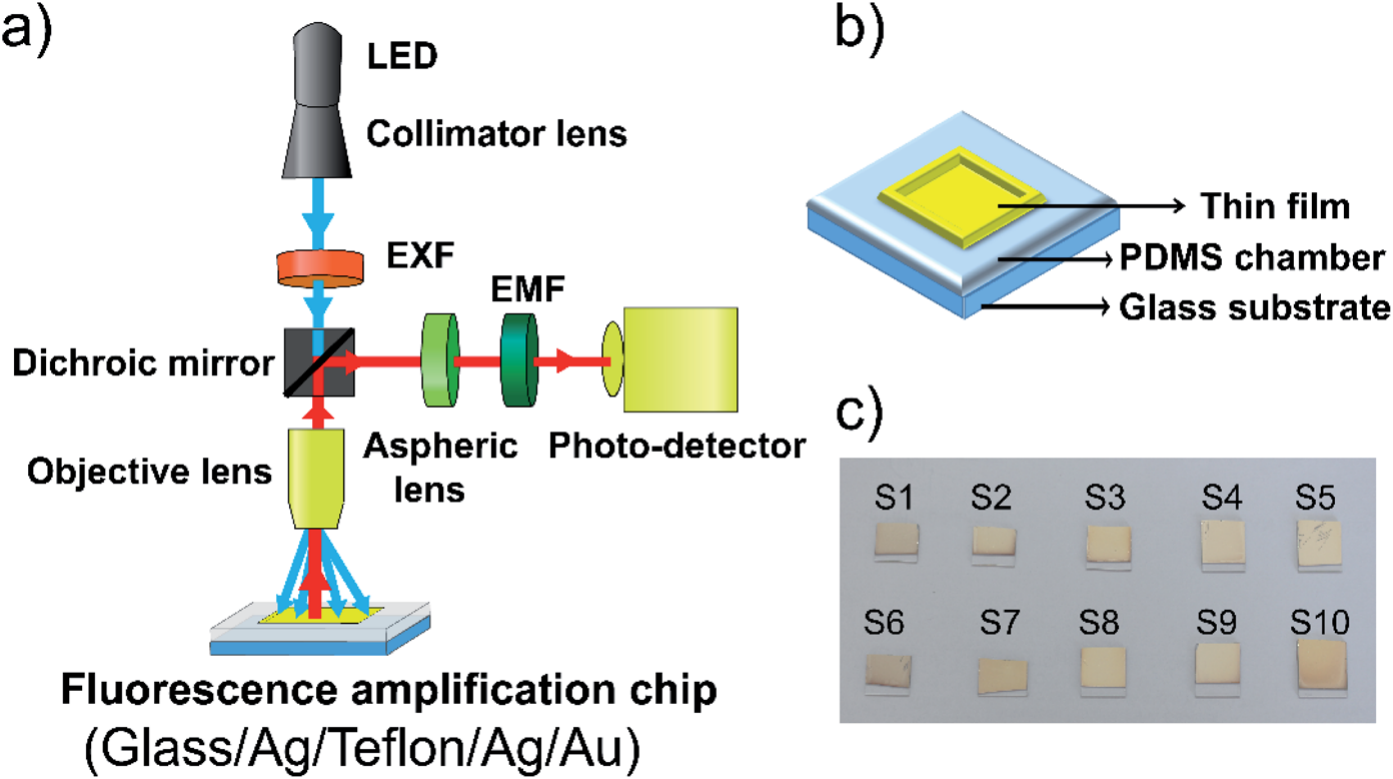
(a) Schematic of the experimental setup used the detect fluorescence emitted into the air, (b) WC-LRSPR sensor with PDMS microfluidic chamber, (c) coating the WC-LRSPR composite film (glass/Ag (40 nm)/Teflon (700 nm)/Ag (5 nm)/Au (5 nm)) with the thermal evaporation system.

We utilized an optical setup with an optical geometry for fluorescence enhancement, as shown in Fig. 2a. Blue excitation light (LED, M470L3-C1, Thorlabs, USA) was transmitted by a filter (ET470/40x, Chroma Technology Corp), dichroic mirror filter, objective lens and excited the sample [Ag(40 nm)/Teflon(650 nm)/Ag(5 nm)/Au(5 nm)]. The emitted green fluorescence passes through (is reflected) by the mirror, filter (ET525/50m, Chroma Technology Corp) and is detected by a photo-detector. We found that fluorescent light from estradiol conjugated with Alexa fluor 488 can be detected at positions both above and below the chip. We adopted to detect light from the upper side of the chip, which results from the radiative coupling of surface plasmon on the metal into the waveguide dielectric side (Teflon) with directional propagation at *λ*_em_. Prior to metal coating, we bonded the PDMS mask with the pristine glass by the oxygen plasma treatment that generates silane bonding (Si–O–Si). This bonding allowed us to use the PDMS mask as a part of a leakage-free microchamber that will contain estradiol receptor antibody conjugated with Alexa fluor 488 (Fig. 2b). For metal deposition (Fig. 2c), we used a thermal evaporation system (BBVL-200-HC12, Japan) operated at a vacuum level of ∼7.3 × 10^−6^ torr, at a deposition rate of ∼4 Å s^−1^ with 7 cm height from the tungsten boat and the glass substrate distance. We used the quartz crystal to monitor the thickness during the evaporating deposition of Ag and Au thin films. For the Teflon layer, we controlled the speed and ratio between PTFE and FC-40 in the spinning coater using the adhesion promoter layer (1*H*,1*H*,2*H*,2*H*-perfluorodecyltriethoxysilane).

Atomic force microscopy (AFM, Hitachi AFM5100N, USA), and FESEM (Hitachi S4800, USA) were used to measure the changing thickness, grain and domain sizes based on the *in situ* monitoring of surface roughness of films. The nanostructures of the fabricated thin films, optical transmittance properties, critical surface tension, and vibrational properties were subsequently studied using a micro-Raman spectrometer (HORIBA XploRA One), PXRD (Bruker D8 Advance diffractometer), UV-vis (V-730/NIR spectrophotometer, JASCO, Tokyo, Japan), a contact angle Phoenix 300 system (Surface & amp; Electro-Optics Co., Ltd), and FTIR (FT-IR spectrometer Frontier from Bruker Vertex 70, Germany), respectively. Fluorescence microscopy analyses (Olympus IX71, Shinjuku, Tokyo, Japan) were performed chemically modified WC-LRSPR surfaces using Alexa fluor 488 labeled goat anti-rabbit IgG (H + L).

## Results and discussion

### Simultaneous excitation of LRSPR and Bi-LRSPR plasmonic structures

**Fig. 3:**
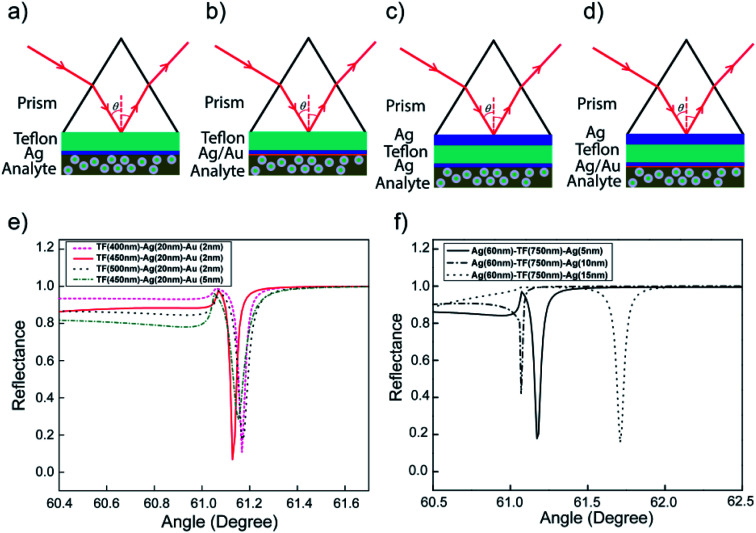
Schematic of various SPR biosensors (a) LRSRP (I-1), (b) Bi-LRSPR (I-2), (c) WCLRSPR (II-1), (d) Bi-WCLRSPR (II-2); (e) effect of materials selection for dielectric Teflon and outer Au layer in the Bi-LRSRP ([prism]–[Teflon]–[Ag]–[Au]); (f) resonance spectra of WCLRSPR configuration with Teflon as a dielectric layer inserted in the two layers of Ag stacks.

Our main focus will be on the resulting symmetric configuration that causes the same surface plasmon frequency to exist on both sides of the Ag layer and occurs between the electromagnetic fields of both surfaces with a variation in the thicknesses of Ag and Teflon. The excitation wavelength is a field at 488 nm in angular interrogation for both the monometallic and bimetallic layers of LRSPR configurations. The metallic thickness optimization has been discussed in our previous analysis for the LRSPR structure.^33^ An LRSPR biosensor (Fig. 3a) was the chosen layer thickness for optimum coupling comprising a prism N-BK7 glass, an Ag upper film (*d*_Ag_ = 15 nm), a Teflon dielectric layer (*d*_Teflon_ = 750 nm) and a liquid with target molecules.^33^Fig. 3 Schematic of various SPR biosensors (a) LRSRP (I-1), (b) Bi-LRSPR (I-2), (c) WCLRSPR (II-1), (d) Bi-WCLRSPR (II-2); (e) effect of materials selection for dielectric Teflon and outer Au layer in the Bi-LRSRP ([prism]–[Teflon]–[Ag]–[Au]); (f) resonance spectra of WCLRSPR configuration with Teflon as a dielectric layer inserted in the two layers of Ag stacks.

For further finer optimization of LRSPR, the excitation of Bi-LRSPR plasmonic itself was undertaken for the inserted Teflon layer, *e.g.* [prism]–[Teflon]–[Ag]–[Au], to give a narrow dip curve in the angular reflectivity spectrum, as shown in Fig. 3b. The dielectric Teflon layer is the typical choice when the dielectric buffer layer needs a refractive index close to that of the intended buffer and is lower than that of the prism. As a consequence, LRSPR refers to a low-frequency coupled mode whose electric field is symmetric on both surfaces. Fig. 3e shows the sensitive response with metamaterial layers providing in terms of the incident angle and minimum reflectance value. Based on the four types of exiting SPR biosensors used for the detection of bio-molecular interaction, the sensitivities and noise limitations were analyzed and compared for angular interrogation and wavelength interrogation. We adjusted the Teflon thickness from 400 to 500 nm to optimize *Γ* for 20 nm of Ag layer thickness and each Au layer of 2 nm and 5 nm. It is obvious that the thinner Au (2 nm) layer gives rise to much narrow minimum dip width and larger penetration depth into the water. The *Γ* of Teflon(450 nm)/Ag(20 nm)/Au(2 nm) configuration gives larger factor of 1.18 times higher than the case (*Γ* = 20.7/°) of the Teflon(750 nm)/Ag(15 nm) structure.

### p-Polarized spectra of WCLRSPR and Bi-WCLRSPR plasmonic structures

This study was on the overall coupling effect of waveguide resonance on the plasmon waves with two plasmonic configurations with a variation in the thickness of the metal (Ag and Au) and dielectric (Teflon), as shown in Fig. 3c and d. Numerical calculation shows that combining LRSPR and bimetallic metal waveguide-coupled SPR of ([Ag1]–[Teflon]–[Ag2]–[Au]) layers can extend the characteristic depth over which the SPR evanescent fields decay at *λ*_ex_. The simulation was carried out for the multilayer ([Ag1]–[Teflon]–[Ag2]) at *λ*_ex_ = 498 nm excitation wavelength, shown in Fig. 3f. For the WCLRSPR finer optimization, the thickness of the inner-Ag layer was fixed to 60 nm, the Teflon waveguide was varied from 650 to 850 nm, and for each of the outer-Ag thickness of 5, 10, and 15 nm. Moreover, FWHM of WCLRSPR is much narrower than the Bi-LRSPR mode as a result of the coupling of the waveguide and plasmonic resonance. Fig. 3 displays the reflectance dip curves; it can be noticed that all the resonance dips of varying thickness of outer-Ag are significantly distinguishable from each other. The use of 750 nm thickness of the Teflon layer provides higher *Γ* than the other Teflon thickness values (650, 750, and 850 nm). Thus, the finer optimized thickness for the outer-Ag thin film can be taken as 5 nm. So a result of tuning of waveguide resonance by increasing the thickness of the waveguide layer, more modes are generated having both plasmonic and waveguide resonance effects. Note that this value of *Γ* calculated by Ag1(60 nm)–Teflon(750 nm)–Ag2(5 nm) is slightly higher by a factor of 1.15 times than the *Γ* = 24.5/° obtained by the Bi-LRSPR coating of the multilayer Teflon(450 nm)/Ag(20 nm)/Au(2 nm).

**Fig. 4:**
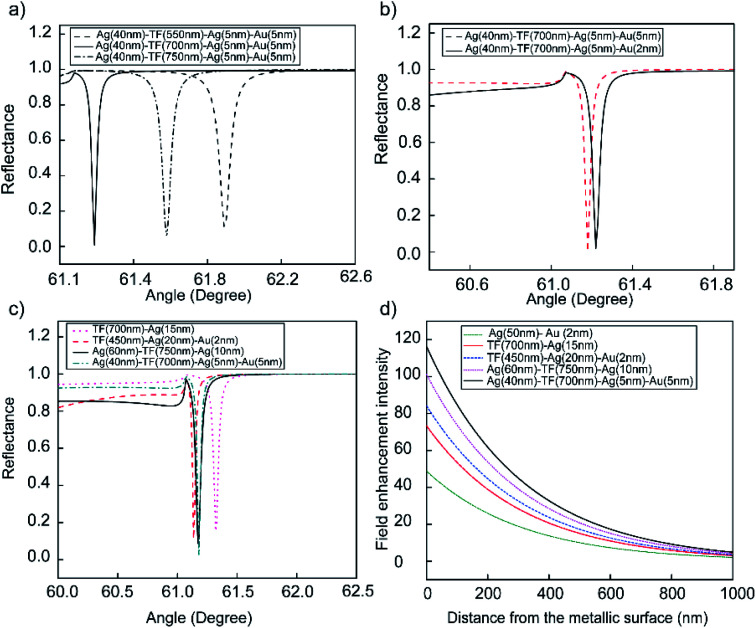
Reflecting spectra as a function of incidence angle with Bi-WCLRSPR configuration. (a) TF (550–700–750 nm), (b) Au (2–5 nm), (c) comparison of the reflection curves *versus* incident angle at different configurations, (d) enhancement of electric field intensity distribution at the surface of the outer metallic layer.

Further increases in *Γ* can be assumed by inserting the Au layer between the outer-Ag and sensing medium layer. Fig. 4a and b show the obtained resonance angle shift as the function of reflectance and the thickness of multilayer of Bi-WCLRSPR in which the Ag–Au bimetallic thin film is used in the LRSPR configuration. To determine an optimized combination of these multilayers, the resonance conditions were analyzed for different Teflon layer thicknesses from 550 to 750 nm, and the fixed thickness of the first layer of Ag, the second layer of Ag and outer-Au thin film were 40, 5, and 5 nm, respectively, as shown in Fig. 4a. When the thickness of Teflon is decreased, the reflectance curve shifts to a lower angle and produces a narrower FWHM in angular interrogation. Here, the thickness of the Teflon layer is chosen as 700 nm for finer optimization.

According to the energy conservation law, if the reflection intensity reaches the minimum value at the resonant angle, the intensity of the electromagnetic field reaches its maximum at the metal surface (*i.e.*, the best coupling coefficient), which can help the best achieved with the SPR technique, high sensitivity,^38^ and resolution.^48,55^ So the final results for tuning with metallic thickness optimization of bimetallic plasmon configuration have also been demonstrated in Fig. 4b. We obtained the incident angle and reflectance of the bi-metallic layer in WCLRSPR as 61.18° and 0.015, respectively. It is clear from Fig. 4b on increasing the thickness of the outer-Au layer from 2 to 5 nm, minimum reflectance increases and gets sharp resonance curves. It may again be noted that the value of *Γ* is slightly higher by a factor of 1.11 times than the *Γ* = 28.3/° obtained by the WCLRSRP coating of Ag(60 nm)/Teflon(750 nm)/Ag(10 nm).

From the above discussion on reflectance dip curves, it can be concluded that the Bi-WCLRSPR model using metal-waveguide-bimetallic multilayer nanostructures provides certain merits over the conventional SPR model. We present in Fig. 4c our simulated results of reflectance SRP dip curves and electric field profiles for p-polarized light to present a comparison between the field profiles of LRSPR with WCLRSPR at the excitation wavelength of (*λ*_ex_ = 488 nm). Specially, we provide a detailed discussion of the ways of optimizing fluorescence enhancement, including the use of a bimetallic layer for enhancing transition rates at spectral channels of excitation (488 nm). Fluorescence enhancement that could be one of the solutions to this challenge can be implemented by Bi-WCLRSPR. We attribute the fluorescence enhancement to both an increase in the fluorophore excitation rate and the elevated rate of the radiative decay from its excited to the ground state. The relevant rates of transition between states involved in fluorophore excitation and emission channels can be modified by SPR, which supports the resonance optical frequency range broad enough to cover both channels. Numerical calculation shows that Bi-WCLRSPR with the bimetallic layer of Au, and Ag as the SPR coating material can extend the characteristic depth over which the SPR evanescent fields decay at *λ*_ex_, increasing the portion of the molecules analyte interacting with plasmonic fields at the spectral channel of the excitation. We find that the Bi-WCLRSPR configuration of Ag(40 nm)/Teflon(700 nm)/Ag(5 nm)/Au(5 nm) multilayer film results in the largest depth-to-width ratio at both *λ*_ex_ and *λ*_em_ among the cases considered. Note that the relative ratio between magnitudes of the real and imaginary parts of *ε*_r_ is much larger in Ag than in Au at *λ*_ex_ and *λ*_em_, which causes the ATR reflectance curve to become narrow in those cases. Such Bi-WCLRSPR configuration provides some interesting results that differ quite significantly from the conventional SPR, and LRSPR structure.

The maximum enhanced electromagnetic (EM) field enhancement *versus* distance from the metallic (Ag, or Au) surfaces with various incidence angles was obtained and plotted in Fig. 4d at *λ*_ex_ = 488 nm. The EM field distributions in the metal film and the dielectric medium are the most important concepts for SPR-sensing since the interaction of the evanescent field with molecules in the sensing region is crucial. Fig. 4d shows the variation of electric field intensity factor with distance from bimetallic layers (Ag(50 nm)/Au(2 nm)) and compared the electric field intensity factor with the single Ag layer and bimetallic Ag/Au on LRSPR and WCLRSPR configuration. As discussed before, the addition of a waveguide layer with the same refractive index of water causes substantial enhancement in the sensor sensitivity. The results clearly show that the Bi-WCLRSPR [Ag(40 nm)/Teflon(700 nm)/Ag(5 nm)/Au(5 nm)] produces larger values of electric field intensity factor than any types of configuration considered, in agreement with results in Fig. 4c. The Bi-WCLRSPR configuration shows the best-optimized E-field intensity (*E* = 116), which is 2.38 times higher than the case (*E* = 48.8) of the bimetallic layers (Ag/Au) of conventional SPR. The results show that the WCSPR [Teflon(450 nm)/Ag(20 nm)/Au(2 nm)] with producing lower than Bi-WCLRSPR by a factor of 1.37.

### Characterization studies

The adhesion-promoter layer was prepared by mixing solutions, ultrapure water, EtOH, and 1*H*,1*H*,2*H*,2*H*-perfluorodecyltriethoxysilane in a weight ratio of 5 : 95 : 1. The adhesion layer was spun at 1500 rpm for 10 s to form a uniform layer on the glass substrate and baked at 100 °C for 10 min. PTFE was diluted with FC-40 to different weight ratios (1 : 2.21), and (1 : 1.17); then spun at different speeds from 2000 to 6000 rpm to determine the appropriate speed for the proper thickness, and baked at 175 °C. AFM was to used measure the film thickness at given the spinning speed.

**Fig. 5:**
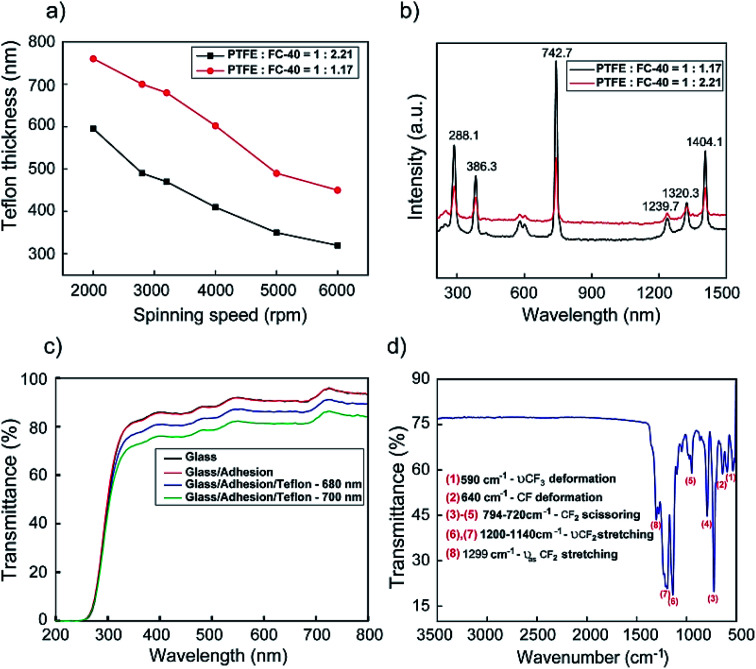
(a) Teflon thickness as a function of spinning speed coating and (b) the Raman spectrum of the Teflon layer with the change of ratio polytetrafluoroethylene (PTFE) and perfluoro(*n*-butyltetrahydrofuran) (FC-40), (c). The optical transmittance of Teflon with adhesion, Teflon film (680, 700 nm) on the glass substrate, (d) IR spectrum of the Teflon film (700 nm thickness) on the silicon wafer.

Fig. 5a shows the speed *versus* thickness for each ratio of dilution (PTFE to FC-40). It can be seen that the thickness of the Teflon coating increase with the reduction of the FC-40 component in the ratio of dilution, from 1 : 2.21 to 1 : 1.17. The Teflon thickness for PTFE : FC-40 = 1 : 2.21 varies from 310 nm to 600 nm and PTFE : FC-40 = 1 : 1.17 from 450 nm to 760 nm. It was the addition of solvent FC-40 that reduced the concentration of PTFE resulting in thinning during spinning. In addition, the difference in spin speed led to differences in layer thickness. Both samples tend to decrease in thickness as the spin speed increases due to more liquids spun out of the substrate during film formation.

From simulation results, we chose the dilute ratio of PTFE : FC-40 (1 : 1.17) for experiments on the Teflon layer by the spinning method. The influence of the distinctive ratio of PTFE : FC-40 on the intensity peaks and the appearance of Raman shifts is implied and presented in Fig. 5b. Specifically, in the Raman spectrum, the signal peaks observed at 288.1 cm^−1^ and 386.3 cm^−1^ can be related to torsional and deformation vibrations of CF_2_, respectively.^56^ The assignment of symmetric, antisymmetric stretching vibration of CF_2_ appeared, respectively, at 742.7 cm^−1^, 1239.7 cm^−1^ and that at 1320.3 cm^−1^, 1404.1 cm^−1^ are due to the stretching vibration of C–C.^56,57^ Aside from this, the results indicated that the intensity of the Teflon layer decreases with the augmentation of FC-40 ratio of PTFE : FC-40 due to the thickness of Teflon formed from PTFE : FC-40 = 1 : 1.17 is thicker than from PTFE : FC-40 = 1 : 2.21.

The comparison of the optical transmittance in the visible range of bare glass, adhesion on glass substrates, and the Teflon film (680, 700 nm) is presented in Fig. 5c. The adhesion film had transparency from 270 to 800 nm with a high transmittance (∼98%) of above 350 nm that was slightly reduced as Teflon was deposited on it. The Teflon thickness of 680 nm (83.4%) demonstrated a higher transmittance value than that of 700 nm (78.6%) at the excitation wavelength of 498 nm. The FT-IR spectrum of the Teflon film with 700 nm thickness on the silicon wafer is shown in Fig. 5d. The peaks observed at 590 cm^−1^, which can arise from the stretching and deforming vibration of CF_3_ groups. Around 640 cm^−1^, the deformation CF bending vibration corresponds to PTFE in the structure of Teflon.^58^ In addition, the other two absorption peaks at 720 and 794 cm^−1^ can be assigned to the scissoring vibration of CF_2_ groups. The strong band at 1140 cm^−1^, along with an additional band at 1200 cm^−1^ in the FT-IR spectrum, is attributed to the symmetric and that at 1299 cm^−1^ to the asymmetric stretching vibrations of CF_2_ groups.

**Fig. 6:**
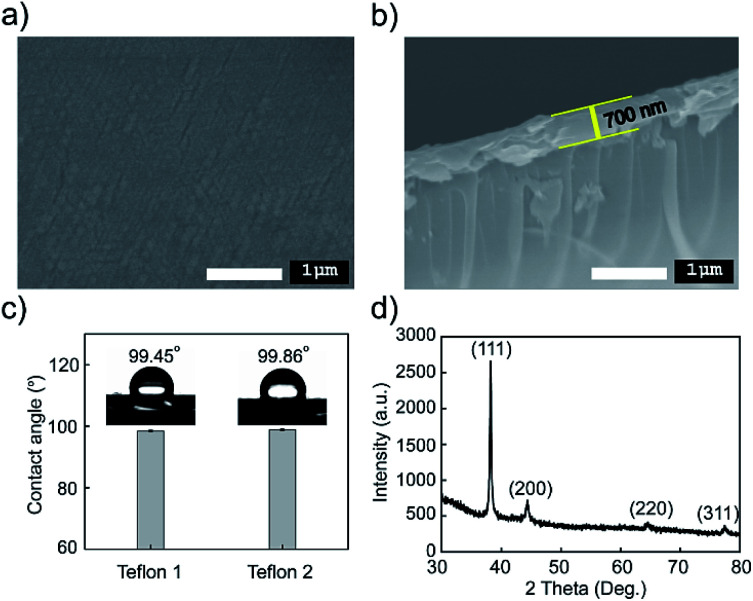
SEM images of the Teflon film (700 nm): (a) surface and (b) cross-section, (c) water contact angle on the surface of Teflon, (d) the XRD pattern of the WC-LRSPR structure glass/Ag/Teflon/Ag/Au film.

Surface morphology and the cross-section of the Teflon film (700 nm) were examined utilizing SEM micrographs as shown in Fig. 6a and b. The black color uniformity and quite low roughness of the Teflon film surface could be observed. Based on the cross-section SEM image, as shown in Fig. 6b, the Teflon layer is clearly distinguishable with the substrate and defines thicknesses of 700 nm. The functionalization on the surfaces of bare glass with the Teflon layer was monitored by water contact angle geometry, as shown in Fig. 6c. There is no existence of water in the FT-IR spectrum of the Teflon film, indicating hydrophobicity that was further proven through water contact angle goniometry. The Teflon film presented strong hydrophobicity through measurement on two different Teflon samples with a contact angle of 99.45° and 99.86°, indicating that the surface wettability was obviously unchanged after numerous fabrications.

The WC-LRSPR structure on the glass/Ag/Teflon/Ag/Au film was analyzed using XRD, as presented in Fig. 6d. The diffraction pattern of the outermost layer Au was recorded displaying well-defined face-centered cubic (FCC) structure with the orientation of (111) at 38°, (200) at 44°, (220) at 64° and (222) at 79°.^59^ This matched with the Au simulated in the database of the Joint Committee on Power Diffraction Standards, USA (JCPDS no. 00-004-0784). The intensity of the (111) peak was much stronger than other peaks that presented preferential growth in the (111) direction of the Au layer in the WC-LRSPR structure. There were no peaks other than the diffraction peaks of Au, verifying that the Au layer was deposited completely purified and tightly covered the surface of the material.

### Microfluidic chip-based optical fluorescence microscopes

**Fig. 7:**
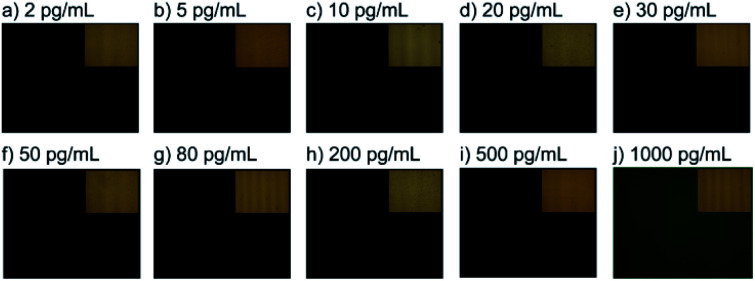
Fluorescence microscopy images of the WC-LRSPR sensors with various concentrations of estradiol (from 2 to 1000 pg mL^−1^). Inset images show the bright-field microscopy image with the white light of these samples.

From the fluorescence microscopy image results (Fig. 7), a WC-LRSPR (glass/Ag/Teflon/Ag/Au) is labeled with a series of concentrations of 17β-estradiol, and then a bright light (excitation light) is used to illuminate the sample, which gives off fluorescence (emission light). As the results show, only the sample with 1000 pg mL^−1^ of 17β-estradiol is highly contrasted on the black background as the fluorophore emits a bright-colored light, which presents antibody molecules on the WC-LRSPR-modified surface compared with the other concentrations. In bright-field microscopy (inset figure), the sample is illuminated with transmitted white light. We could clearly see the structure of the sample under the microscope with high contrast.

### Fluorescence detection and enhancement factors

**Fig. 8:**
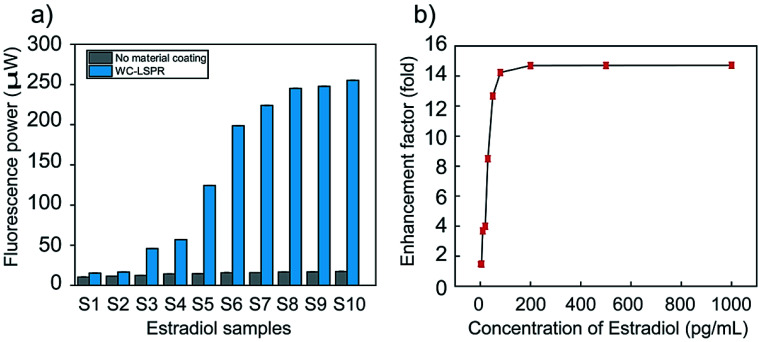
(a) Estimated values of fluorescence optical power *versus* the concentration, (b) fluorescence enhancement factors experimentally obtained by the optical setup with the dielectric waveguide (Teflon) on the SPR sensor.

In order to estimate the factor of enhancement of fluorescence detected, we considered two cases in the experiment for fluorescence enhancement, *i.e.*, one with metal coating and the other without any Ag/Teflon/bimetallic coating (Fig. 8). We use deionized water as a liquid buffer for fluorophores and found that it fluoresces little, producing negligible autofluorescence. All experiments were repeated four times to obtain standard error and coefficient of variation (CV). In estimating the optical power of pure fluorescence, which must be used for fluorescence enhancement analysis, dark noise power included in the detected fluorescence power needs to be subtracted.^60,61^Fig. 8 shows the fluorescence optical power measured *via* the optical systems described in Fig. 2a. First, it was found that the use of 2 pg mL^−1^ of 17β-estradiol (CV < 1%), *i.e.*, the use of Ag/Teflon/bimetallic coated-slide glass enhances the fluorescence as compared to that achieved with no metal coating by 1.47-fold (Fig. 8b).

Compared to related studies of SPR detection technique-based detection of estradiol (Table 1S†), the LOD at 2 pg mL^−1^ was verified for the enhancement efficiency of the synthesized Bi-WCLRSPR chip. Furthermore, we can afford to enhance fluorescence further by a factor of up to 14.7-fold by using 1000 pg mL^−1^ of 17β-estradiol in the reflection configuration. It is also revealed that, in the reflection mode, the chip of the Ag/Teflon/bimetallic-coated glass slide allows higher enhancement of fluorescence to be achieved, *i.e.*, 1.47-fold, which we can attribute to the SPR evanescent field decay depth longer than that of the no metal-coated slide glass chip at *λ*_em_. The dependence of fluorescence enhancement on the decay depth of plasmonic evanescent fields signifies that fluorophores were sufficiently far away from the metal surface making little coupling with surface plasmons and their normal fluorescence is effectively blocked by the metal layer coated on a glass slide, a negligible amount of fluorescent light being detected by a photo-detector. This is in contrast to the fluorophores in close proximity, which makes near-field coupling with surface plasmons into substantial radiative luminance below the metal-coated glass slide. Interestingly, fluorescence saturation with higher estradiol concentration from 200 to 1000 pg mL^−1^ is observed. It follows that fluorophores interacting with surface plasmons are governed by photobleaching, leading to an extended scale of saturation between the concentration of the estradiol conjugated fluorophores and fluorescence intensity. This bleaching feature of fluorophores interacting with surface plasmons accounts for a saturate enhancement factor with a concentration of the 17β-estradiol template.

## Conclusions

In conclusion, a methodology is obtained from the enhanced EM fields in WCLRSPR by exciting the waveguide and surface plasmon resonance on bimetallic films. Due to the strong EM field enhancement and the large SPR dip curve of Bi-WCLRSPR, the improvement and optimization of the sensor based on Ag(40 nm)/Teflon(700 nm)/Ag(5 nm)/Au(5 nm) multilayer film coating was produced. This sensor showed more than 2.38 times enhanced electric field intensity factor, high sensitivity, and overlap with the SPR reflectance condition. It is visible that the use of the application chip with metallic coating for Bi-WCLRSPR enhances the fluorescence as compared to that achieved with no metal coating, in reflection configuration. The present calculation is used to make the proposed dielectric waveguide (Teflon) on the SPR sensor will open a new window in high-performance sensing. This study highlighted the great potential of WC-LRSPR for the development of biosensors. The results are consistent with our proposed model of the coupling and transferring to the excited state energies of fluorophores to Au surface plasmons. Concluding this study, the developed WC-LRSPR technique allowed for robust and simple detection of the minimum detectable concentration of 2 pg mL^−1^ estradiol (CV < 1%) within a reaction time of 5 min. The fluorescence signals (from 2 to 1000 pg mL^−1^) can be monitored in real-time, which allowed for advanced biomolecular interaction analysis. It has been reported that the degree of enhancement in the fluorescence intensity was 14.7-fold at 1000 pg mL^−1^ concentration estradiol. Our study suggests a significant opportunity for developing of sensors for detecting of female sex steroid hormones for diverse applications in life sciences.

## Conflicts of interest

There are no conflicts to declare.

## Supplementary Material

RA-011-D1RA02130C-s001
