# Supplementary material for: Ultrasensitive biosensors based on waveguide-coupled long-range surface plasmon resonance (WC-LRSPR) for enhanced fluorescence spectroscopy
Source: RSC Adv. 2021 Jun 25;11(36):22450–60. doi: 10.1039/d1ra02130c (PMC9034228; doi:10.1039/d1ra02130c)
Supplement: RA-011-D1RA02130C-s001 [file RA-011-D1RA02130C-s001.pdf]

Electronic Supplementary Material (ESI) for RSC Advances.
This journal is © The Royal Society of Chemistry 2021

Supplementary Materials

Ultrasensitive biosensors based on waveguide-coupled long-range surface plasmon resonance (WC-LRSPR) for enhanced fluorescence spectroscopy

Nhu Hoa Thi Tran,[a,b,*] Viet-Duc Phung,[c,d] Hanh Kieu Thi Ta,[a,b,e] Vu Dinh Lam,[f] Do Hung Manh,[g] Ngoc Kim Pham,[a,b,e] Jae Young Kim,[h] Nae Yoon Lee,[i] Bach Thang Phan[b,e,j,]**

[a] Faculty of Materials Science, University of Science, HoChiMinh City, Viet Nam.
[b] Vietnam National University, HoChiMinh City, Viet Nam.
[c] Future Materials and Devices Laboratory, Duy Tan University, Ho Chi Minh City, 700000, Viet Nam.
[d]Faculty of Environmental and Chemical Engineering, Duy Tan University, Da Nang, 550000, Viet Nam.
[e] Center for Innovative Materials and Architectures (INOMAR), HoChiMinh City, Viet Nam.
[f] Graduate University of Science and Technology, Vietnam Academy of Science and Technology, Hanoi, Vietnam.
[g] Institute of Materials Science, Vietnam Academy of Science and Technology, Hanoi, Vietnam.
[h]Department of Life Science, Gachon University, Seongnam-si, Gyeonggi-do 13120, Republic of Korea.
[i] Department of BioNano Technology, Gachon University, Seongnam-si, Gyeonggi-do 13120, Republic of Korea.
[j] Laboratory of Advanced Materials, University of Science, HoChiMinh City, Viet Nam.
E-mail: ttnhoa@hcmus.edu.vn, and pbthang@inomar.edu.vn

Table 1S: Comparison of the LOD values for estradiol immunoassays reported publication

Method	Chip	LOD	Ref
SPR (inhibitive assay)	Gold sensor	200 pg/mL	Miyashita et al.[1]
Colorimetric detection	Gold nanoparticles	200 pM	Alsager et al.[2]
SPR (inhibitive assay)	3-MPA/E2/OEG/CM5 sensor chip	25 pg/mL	Mitchell et al.[43]

References

1 M. Miyashita, T. Shimada, H. Miyagawa and M. Akamatsu, *Anal. Bioanal. Chem.,* 2005, **381**, 667–73.

2 O. A. Alsager, S. Kumar, B. Zhu, J. Travas-Sejdic, K. P. McNatty and J. M. Hodgkiss, *Anal. Chem.* , 2015, **87**, 4201–9.

43 J. S. Mitchell, Y. Wu, C. J. Cook and L. Main, *Steroids*, 2006, **71**, 618–631.
